# Feasibility of a Smoking Cessation Smartphone App (Quit with US) for Young Adult Smokers: A Single Arm, Pre-Post Study

**DOI:** 10.3390/ijerph18179376

**Published:** 2021-09-05

**Authors:** Phantara Chulasai, Dujrudee Chinwong, Surarong Chinwong, John J. Hall, Purida Vientong

**Affiliations:** 1 PhD’s Degree Program in Pharmacy, Faculty of Pharmacy, Chiang Mai University, Chiang Mai 50200, Thailand; panthara.chula@gmail.com; 2 Department of Social Pharmacy, Faculty of Pharmacy, Payap University, Chiang Mai 50000, Thailand; 3 Department of Pharmaceutical Care, Faculty of Pharmacy, Chiang Mai University, Chiang Mai 50200, Thailand; dujrudee.c@cmu.ac.th (D.C.); surarong@gmail.com (S.C.); 4 Cluster of Excellence on Biodiversity-Based Economic and Society (B.BES-CMU), Chiang Mai University, Chiang Mai 50200, Thailand; 5 School of Population Health, University of New South Wales, Sydney 2052, Australia; john.hall@unsw.edu.au

**Keywords:** smartphone, mobile phone, smartphone application, smartphone app, mobile app, app, smoking cessation, young adults, young adult smokers

## Abstract

While smartphone applications (apps) have been shown to enhance success with smoking cessation, no study has been conducted among young adult smokers aged 18–24 years in Thailand. Quit with US was developed based on the 5 A’s model and self-efficacy theory. This single arm, pre-post study was conducted aiming to assess results after using Quit with US for 4 weeks. The primary outcome was a biochemically verified 7-day point prevalence of smoking abstinence. The secondary outcomes included smoking behaviors, knowledge and attitudes toward smoking and smoking cessation, and satisfaction and confidence in the smartphone app. A total number of 19 young adult smokers were included; most participants were males (68.4%) with the mean (SD) age of 20.42 (1.46) years. After 4 weeks of study, the primary outcome demonstrated a smoking cessation rate of 31.6%. All 19 participants expressed better smoking behaviors and better knowledge and attitudes toward smoking and smoking cessation. Further, they were satisfied with the smartphone app design and content and expressed confidence in using it. These findings provided preliminary evidence that Quit with US was found to be a potentially effective smoking cessation smartphone app for young adult smokers.

## 1. Introduction

Effective smoking cessation interventions among young adult smokers aged 18–24 years are invaluable because they have distinctive smoking behaviors from adult smokers. Their smoking behaviors are more influenced by behavioral and social factors [1,2]. The smoking cessation intervention that is the most effective for them has not been conclusively elucidated yet. Even though young adult smokers express interest and determination to quit smoking, their smoking cessation rate remains lower than that of adult smokers [3]. For those attempting to quit smoking, very few sought consultations from medical professionals [4,5,6].

Thailand has implemented various tobacco control policies in compliance with the WHO Framework Convention on Tobacco Control, which has shown remarkable success in declining overall smoking rates over the past two decades [7,8,9]. However, challenges remain for young adult populations. The smoking rate among university students in Chiang Mai Province, located in northern Thailand, was 14.5% in 2006 [10] and the national smoking rate among young adults gradually increased from 19.9% in 2013 to 20.4% in 2017 [9]. This suggests that more comprehensive and effective tobacco control policies for young adult populations are necessary. Expected to strengthen the reduction of young adult smoking, several tobacco control regulations have been legislated. For instance, the minimum age for purchasing tobacco products has been raised from 18 to 20 years and smoking in all educational institutions or places of learning and training is prohibited [7,11]. Therefore, identifying an effective smoking cessation intervention is particularly valuable for young adult smokers to help them quit smoking and reduce the smoking rate further.

Studies on smoking cessation methods among young adult smokers remain limited. Even though community pharmacist counseling on smoking cessation was deemed effective as an intervention recommended in several national guidelines [12,13], most counseling clients were either elderly subjects with chronic diseases or prone to experiencing health problems and a scalability of smoking cessation counseling provided in community pharmacy is still limited [14]. Interactive health education tools available on smartphone apps have demonstrated increase knowledge, skills, and self-efficacy levels among patients with various health conditions, including smoking cessation [15]. The development of smoking cessation methods using smartphone-based platforms tends to improve the success rate in quitting smoking for young adult smokers [16] because the current generation of young adults spends their daily lives interacting with various digital technologies and is the highest-ranked user of the Internet via mobile smartphones [17]. 

Currently, many smartphone apps are devised to assist with smoking cessation. However, less than one-third of the smartphone apps presented a high level of content adherence to smoking cessation treatment guidelines [18,19]. No smartphone apps contain the entire content of the 5 A’s model in guidelines for smoking cessation: ask, advise, assess, assist, and arrange [20]. Recent randomized controlled trials (RCT) studying smartphone apps for smoking cessation among adult smokers demonstrated conflicting findings when compared with a controlled group indicating both negative and positive outcomes of the smartphone app use regarding smoking cessation rate [21,22,23,24,25,26,27,28,29]. While some RCTs are still unpublished [30,31], only one finished RCT was conducted among young adult smokers in Canada [32]. Its findings revealed that the smartphone app neither increased the smoking cessation rate nor reduced smoking consumption when compared with a printed self-help guide. However, the evaluation results regarding the use of this studied smartphone app revealed significantly higher levels of overall satisfaction and perceived helpfulness.

Limited understanding remains regarding smartphone apps developed to support smoking cessation for young adult smokers in Thailand. This led to the development of Quit with US, a novel smartphone app for smoking cessation among young adult smokers aged 18–24 years. Quit with US was designed and developed to be used alone or combined with existing smoking cessation methods, including counseling by pharmacists, to promote successful smoking cessation. The smartphone app was invented following smoking cessation treatment guidelines with especially focusing on the 5 A’s model [12,13] and self-efficacy, a theory of individuals’ confidence in their ability to perform specific health behavior [33]. Greater self-efficacy in smoking cessation was reliably associated with successful smoking abstinence [34,35,36,37] and a decrease in relapse smoking [38].

After the smartphone app had been designed and developed, this study was then conducted aiming to assess preliminary results following the smartphone app use including smoking cessation, smoking behaviors, knowledge and attitudes toward smoking and smoking cessation, and satisfaction and confidence in the use. The findings of this study could be further used as guidance to improve this smartphone app. After that, the improved version of Quit with US will be assessed for its efficacy in a randomized control study.

## 2. Materials and Methods

### 2.1. Study Design and Participants

This single arm, pre-post study was conducted between October and December 2019 in Chiang Mai Province, Thailand. A total of 19 undergraduate smokers, selected using a purposive sampling method, voluntarily participated in this study. The inclusion criteria were as follows: (1) 18–24 years old; (2) history of smoking at least one cigarette in the last 30 days; (3) interest in quitting smoking; (4) possession of smartphones with Android operating system supporting the smartphone app use; and (5) ability to communicate in Thai. Participants received 400 Thai baht (approximately US $12.4) as compensation for each visit.

### 2.2. Quit with US

A research team developed Quit with US in cooperation with a programmer team aiming to obtain a smartphone app encouraging young adult smokers to achieve smoking cessation. The development process of the smartphone app consisted of three sequential steps. Step 1: conceptual and theoretical framework regarding the 5 A’s model recommended in treatment guidelines for smoking cessation [12,13], and concepts and various theories including self-efficacy [33] were explored. A smartphone app framework was conceptualized specially focused on the 5 A’s model. The 5 A’s model comprised five major steps to encourage successful smoking cessation: (1) ask about tobacco use status, (2) advise to quit smoking, (3) assess willingness to quit smoking, (4) assist smokers with a personalized quit plan, and (5) arrange a follow-up contact to monitor smoking cessation.

Step 2: a smartphone app prototype was designed and developed from the smartphone app framework that was conceptualized in Step 1. It subsequently underwent a usability test where its content and confidence in the smartphone app prototype were assessed by pharmacists specialized in advising on smoking cessation. This was conducted in parallel with evaluating the opinions and confidence levels in the smartphone app prototype from undergraduate smokers. During these early stages of development, the smartphone app design and content were particularly addressed to ensure appropriate use for young adult smokers. Lastly, Step 3: the smartphone app was designed and developed employing the evaluation outcomes of the smartphone app prototype. The preliminary results of the developed smartphone app were assessed in this study. As for this preliminary study, the smartphone app was specifically developed for the Android operating system in compliance with Google Play policies.

**Figure 1:**
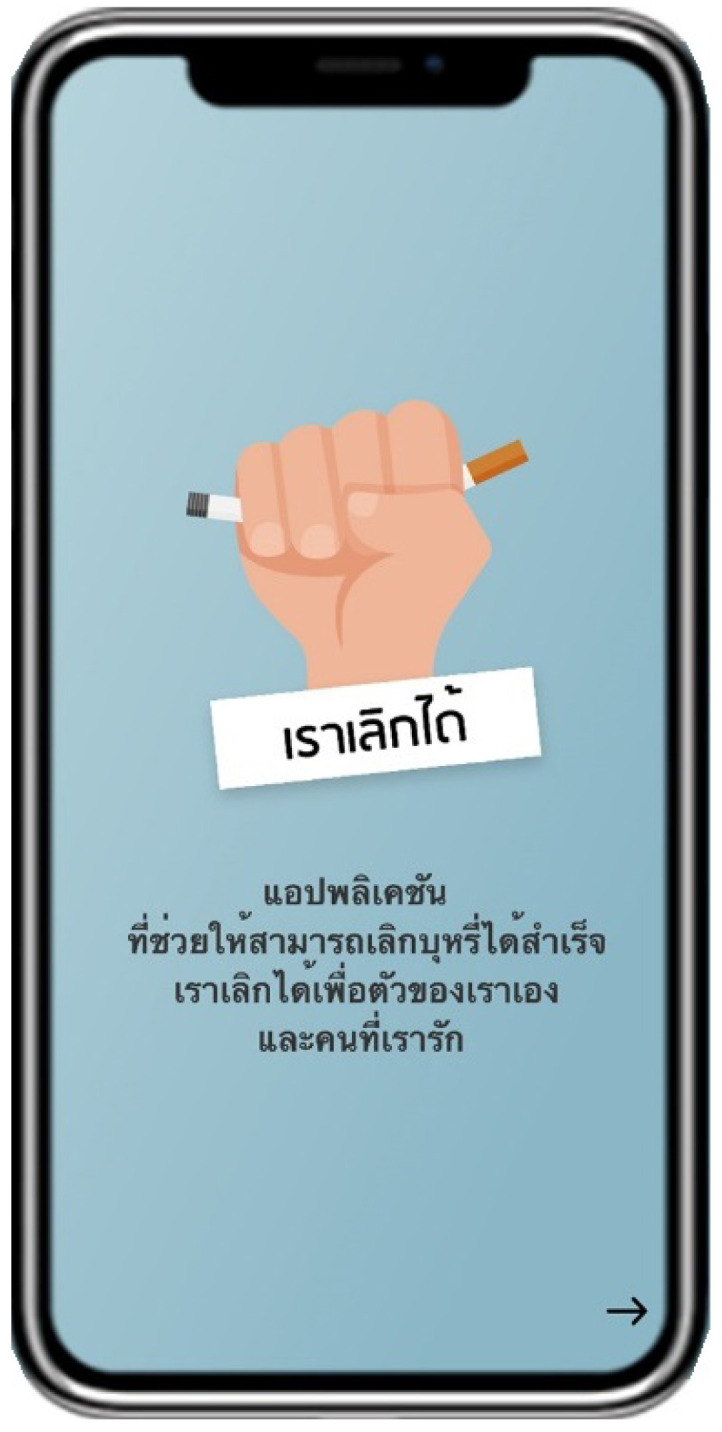
Logo of Quit with US.

Quit with US (Figure 1) is a smartphone app providing smoking cessation assistance in Thai. Its displayed presentations are individualized based on users’ input data. For the first time entering the smartphone app, it asks about tobacco use status and smoking behaviors (for instance daily cigarette consumption, time to the first cigarette of the day, smoking time, and readiness to quit smoking). The smartphone app consists of five main pages, as described below.

**Figure 2:**
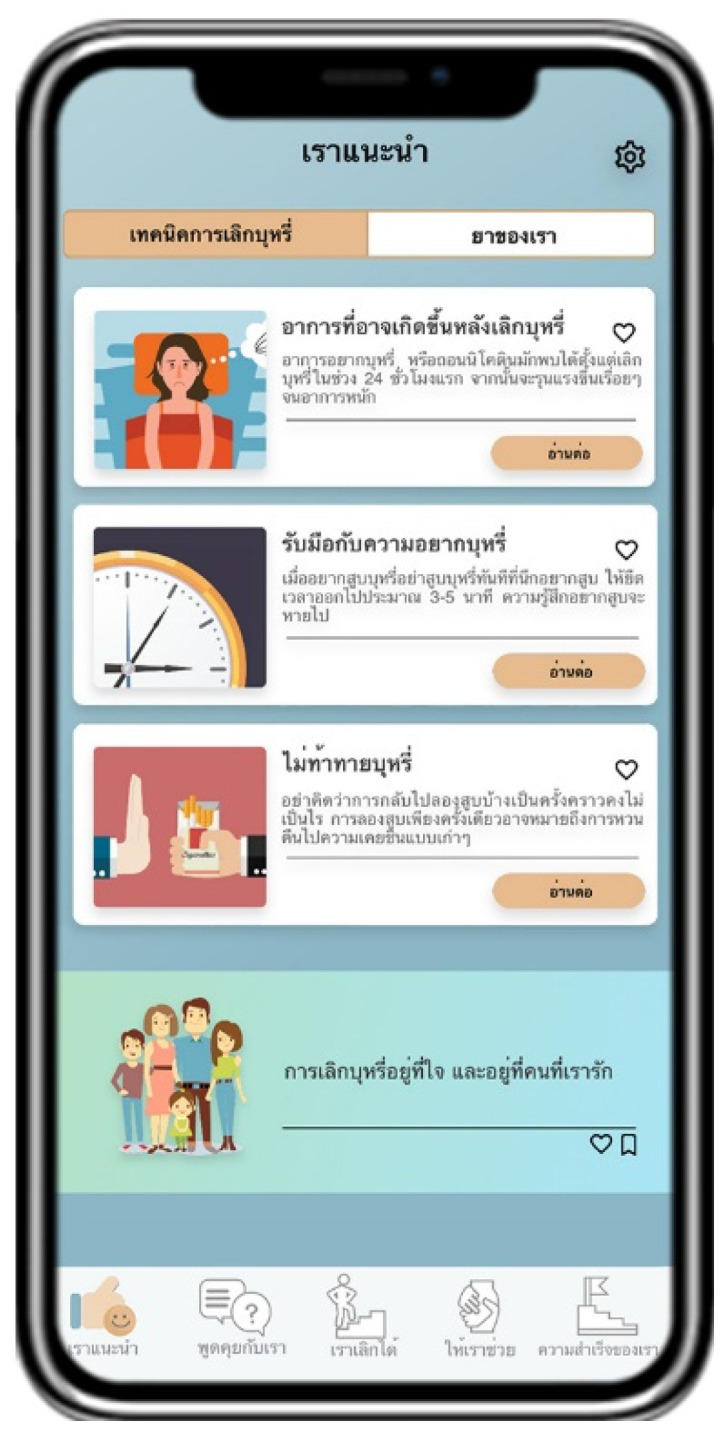
Display screen of Suggested by US.

**Figure 3:**
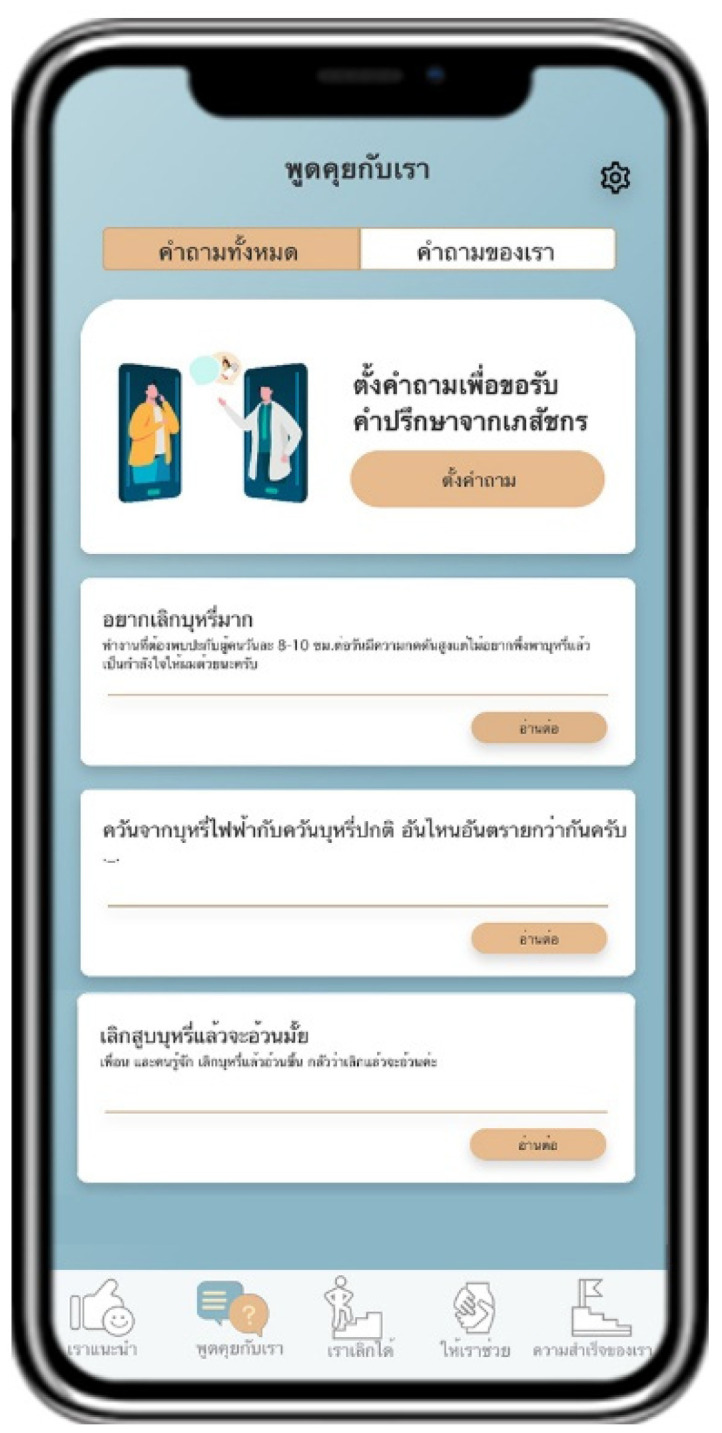
Display screen of Talk with US.

**Figure 4:**
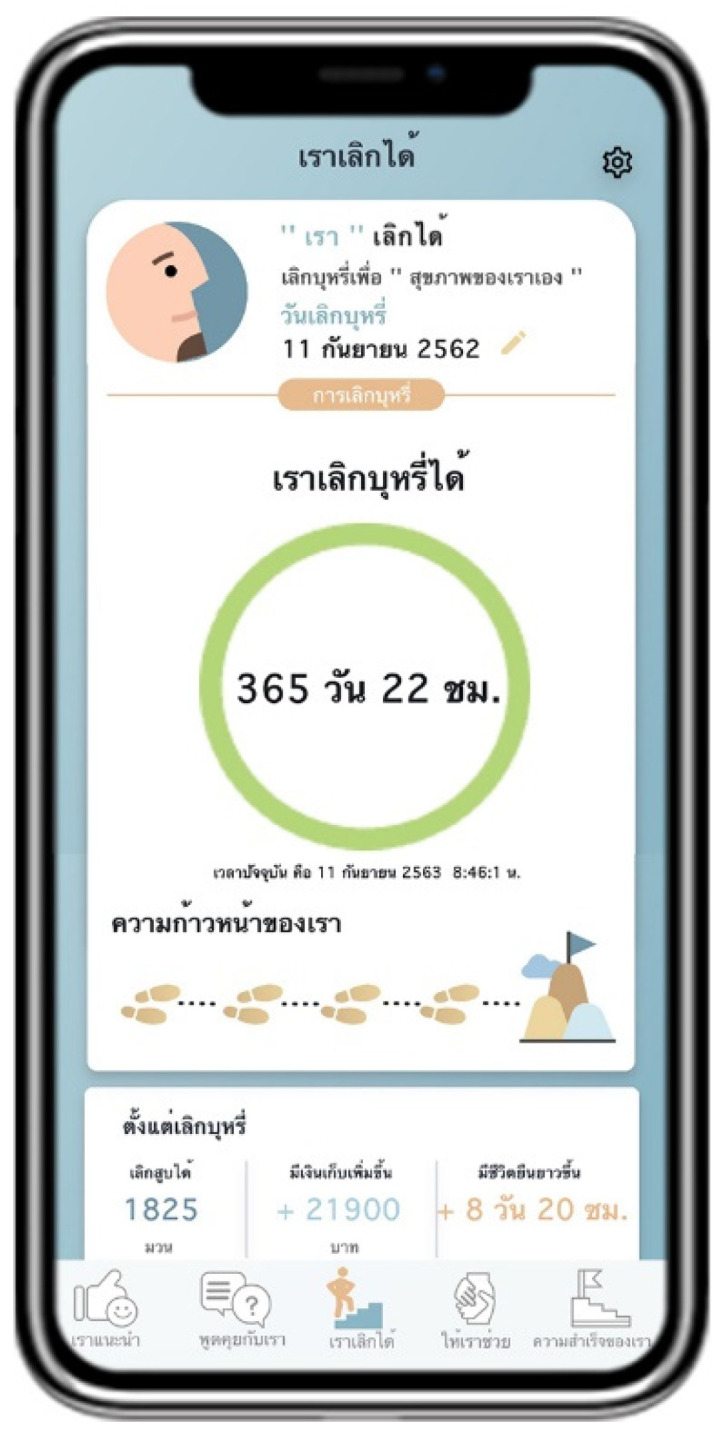
Display screen of Quit with US.

**Figure 5:**
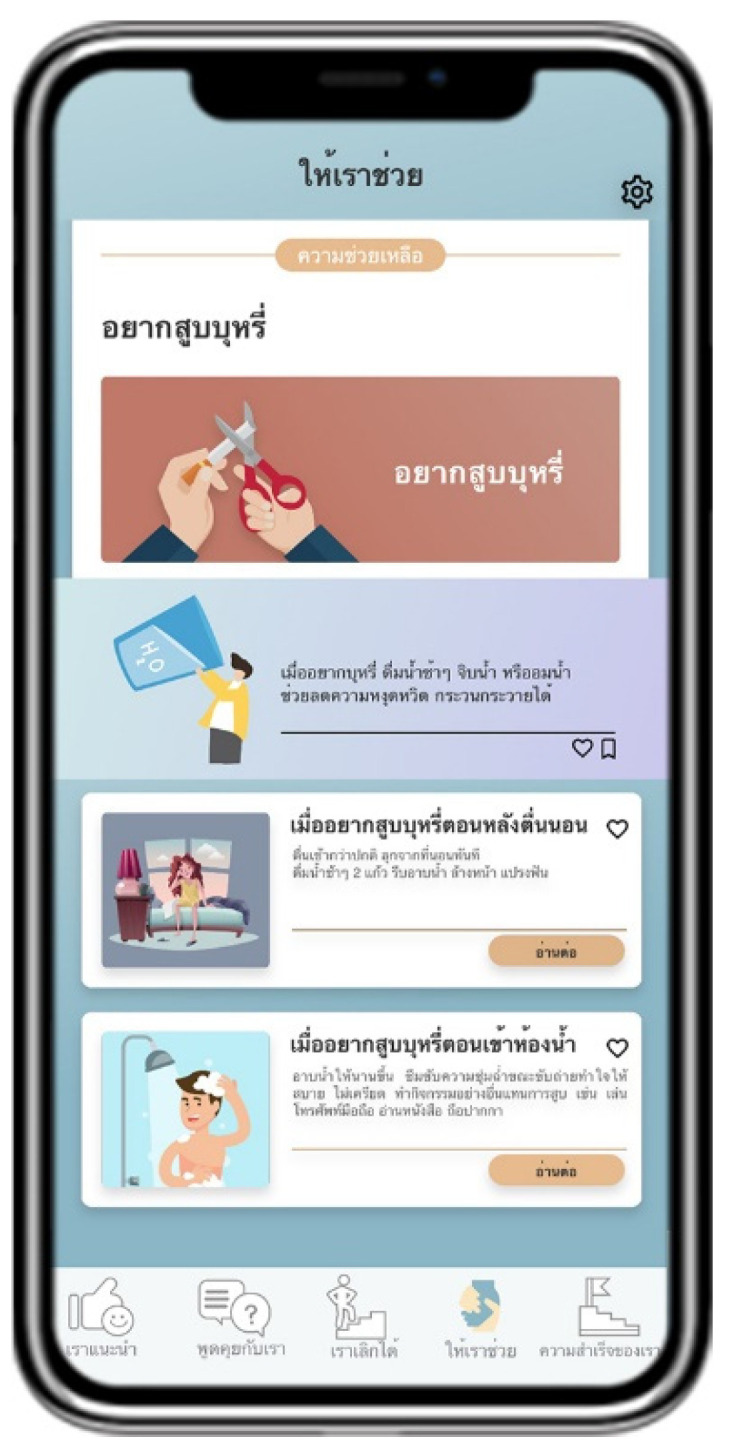
Display screen of Let US Help.

**Figure 6:**
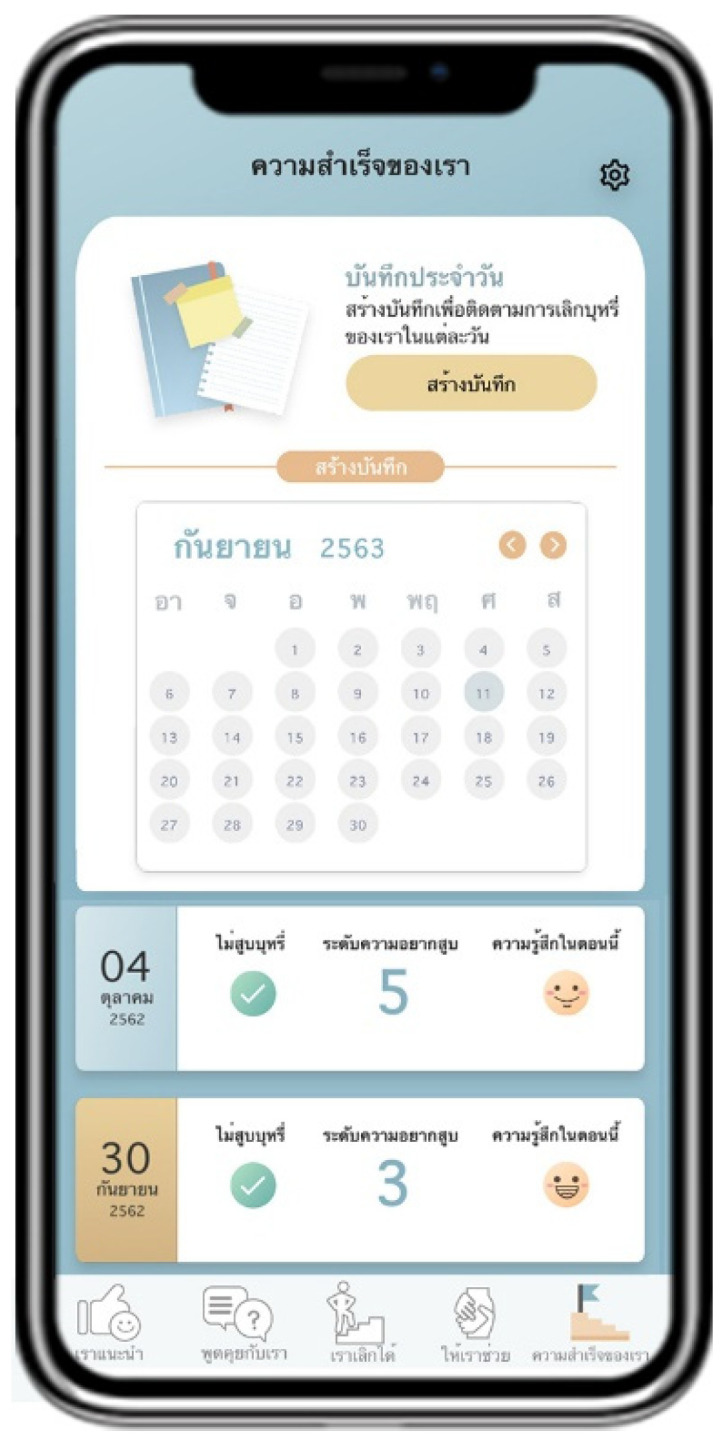
Display screen of Success of US.

Suggested by US advises users to quit smoking by providing information about the pros and cons of smoking and introducing information about smoking cessation methods (Figure 2). Talk with US arranges a follow-up channel to encourage smoking cessation by containing a list of questions and answers provided by pharmacists (Figure 3). Quit with US assesses and displays the cause of tobacco dependence and users’ readiness to quit smoking. This page also assists users with a personalized quit plan by displaying a purpose and target date for quitting smoking set by individual users (Figure 4). Let US Help assists smoking cessation by suggesting coping methods when having symptoms after smoking cessation, desire to smoke, or unintentional smoking (Figure 5). The success of US arranges a self-monitoring of smoking cessation by displaying a smoking diary and tracking information on the progress in quitting smoking recorded by individual users (Figure 6).

### 2.3. Study Procedure

This study was conducted in a counseling area of a university-affiliated pharmacy of the Faculty of Pharmacy, Chiang Mai University. Participants were invited to visit a research team twice throughout the 4-week study. Eligible participants were thoroughly informed on all details of the study appearing in the subject information sheets and required to sign informed consent forms before entering the study. On the first day of the study, all participants were asked to complete self-administered baseline questionnaires consisting of three parts including participants’ general information, smoking behaviors, and knowledge and attitudes toward smoking and smoking cessation. After that, they performed a breath test using piCO Smokerlyzer [39] to measure their exhaled carbon monoxide (CO) concentration level. Next, participants received face-to-face brief counseling on smoking cessation from an experienced pharmacist on the research team who had been trained and certified by the Medical Association of Thailand. The smoking cessation consultation was undertaken thoroughly using the counseling manual written by the research team in accordance with national smoking cessation treatment guidelines (Table S1) [13] and lasted between 10 and 15 min. After the participants downloaded and installed Quit with US completely, they were given advice on using Quit with US and reminded to use the smartphone app at least once a day throughout the 4-week study.

After using Quit with US for 4 weeks, all participants were invited to visit the research team again to complete self-administered follow-up questionnaires and to measure their exhaled CO concentration level. Follow-up questionnaires consisted of three parts including smoking cessation and smoking behaviors, knowledge and attitudes toward smoking and smoking cessation, containing a list of items identical to those in the baseline questionnaire, and opinions toward the smartphone app used to evaluate users’ satisfaction and confidence.

The questionnaires were developed following a literature review of relevant studies. The content validity was evaluated by three experts to assess whether the question items were representative of all research objectives. The Index of Item-Objective Congruence (IOC) score was then identified. The items having IOC scores higher than or equal to 0.5 were considered appropriate; those having IOC scores less than 0.5 were considered inappropriate and were revised regarding recommendations from the experts. The questionnaires were piloted by 31 undergraduate smokers to assess the reliability and the use of appropriate language. The internal reliability test by Cronbach’s alpha measurement revealed a coefficient of 0.769 and 0.739 for items regarding knowledge and attitudes toward smoking and smoking cessation, respectively. Finally, the modified questionnaires with acceptable accuracy and appropriateness were used in this study.

### 2.4. Measures

#### 2.4.1. Primary Outcome

The smoking cessation outcome was evaluated in the fourth week after enrollment using a 7-day point prevalence abstinence, a measurement standard in smoking cessation trials, which is defined as a continuous abstinence from smoking in the previous 7 consecutive days [40]. This measurement was performed by collecting data of self-reported smoking abstinence and subsequently verifying the obtained data with an exhaled CO concentration level; a cut-off level of ≤6 parts per million (ppm) for successful abstainers [39].

#### 2.4.2. Secondary Outcomes

Secondary outcomes were measured to evaluate the following three aspects: (1) smoking behavior, (2) knowledge and attitudes, and (3) satisfaction and confidence.

The smoking behavior outcomes were evaluated by comparing daily cigarette consumption and nicotine dependence level, using the Heaviness of Smoking Index (HSI) score, before and after the use of Quit with US for 4 weeks. The overall HSI score ranged between 0 and 6. This score was calculated from two questions in the questionnaire using summative assessment, namely, time of the first cigarette of the day (≤5 min, 3 scores; 6 to 30 min, 2 scores; 31 to 60 min, 1 score; and ≥61 min, 0 scores) and daily cigarette consumption (1 to 10 cigarettes, 0 scores; 11 to 20 cigarettes, 1 score; 21 to 30 cigarettes, 2 scores; and ≥31 cigarettes, 3 scores) [41].

The outcomes concerning knowledge and attitudes toward smoking and smoking cessation were evaluated and compared before and after using Quit with US for 4 weeks. The evaluation of knowledge on smoking and smoking cessation consisted of 15 question items. These question items were answered by either true or false. Scores were calculated based on the answers: incorrect answer, 0 scores; and correct answer, 1 score. The total cumulative score ranged between 0 and 15, with high scores indicating better knowledge on smoking and smoking cessation. Likewise, the evaluation of attitudes toward smoking and smoking cessation consisted of 15 question items. Scores were calculated based on the answers to each attitudinal statement: disagree, 1 score; not certain, 2 scores; and agree, 3 scores. The answer to negative-meaning question items were inverted to calculate the scores. The total cumulative score ranged between 15 and 45, with high scores indicating positive attitudes toward smoking and smoking cessation.

The satisfaction and confidence in smartphone app outcomes were evaluated in the fourth week after the smartphone app use using a total number of 20 question items for satisfaction evaluation (9 items regarding design and 11 items regarding content) and 8 items for confidence evaluation. All the above question items were rated on a 5-point scale from 1 (the lowest) to 5 (the highest), scores were then calculated by averaging participants’ answers to each item. The items regarding satisfaction and confidence were developed based on the Technology Acceptance Model (TAM) [42] and the System Usability Scale (SUS) [43]. TAM is a well-recognized model describing users’ acceptance and intention to use smartphone apps while SUS is a reliable tool to evaluate the usability of the smartphone apps.

### 2.5. Statistical Analysis

Descriptive statistics for categorical variables were reported as frequency and percentage, while continuous variables were reported as means and standard deviation (SD) or median and interquartile range (IQR) as appropriate. Wilcoxon signed-rank test was used to compare differences between before and after using Quit with US of continuous outcomes regarding exhaled CO concentration level, daily cigarette consumption, HSI score, and rated scores of knowledge and attitudes toward smoking and smoking cessation. Analyses were conducted using Stata 14 Software (StataCorp LP, 2015, College Station, TX, USA). Statistical significance in this study was defined as two-tailed and the alpha level of 0.05.

## 3. Results

### 3.1. Participants’ Characteristics

According to the data gained from all 19 participants, the mean (SD) age was 20.42 (1.46) years. The majority of participants were males (68.4%), identified as daily smokers (68.4%), smoking less than or equal to 5 cigarettes daily (52.6%), and at a low nicotine dependence level evaluated by HSI score (79.0%). Most participants had a history of quit attempts during the past year (79.0%), but no participants had experienced using neither Thai nor English smartphone apps to assist with their smoking cessation (Table 1).

### 3.2. Evaluating Outcomes

#### 3.2.1. Smoking Cessation

After using Quit with US for 4 weeks, the smoking cessation was evaluated using a self-reported 7-day point prevalence abstinence with subsequent verification by exhaled CO concentration level. Six of the 19 participants (31.6%) achieved smoking cessation. Moreover, the mean (SD)median (IQR) exhaled CO concentration level of all participants significantly decreased from 9.47 (4.97) ppm [median (IQR) = 8 (6, 12)] at baseline to 7.84 (3.53) ppm [median (IQR) = 8 (4, 11)] in the fourth week (*p* = 0.014) (Table 2).

#### 3.2.2. Smoking Behaviors

All participants expressed better smoking behaviors. The results demonstrated that their mean (SD) daily cigarette consumption significantly decreased from 7.05 (5.66) [median (IQR) = 5 (3, 10)] at baseline to 4.00 (4.80) [median (IQR) = 3 (0, 5)] in the fourth week (*p* = 0.008). Likewise, the mean (SD) HSI score also significantly decreased from 1.16 (1.46) [median (IQR) = 0 (0, 2)] to 0.42 (0.69) [median (IQR) = 0 (0, 1)] (*p* = 0.010) (Table 2).

Regarding 13 nonabstainers who failed to quit smoking, their mean (SD) daily cigarette consumption significantly decreased from 9.31 (5.48) [median (IQR) = 7 (5, 10)] to 5.85 (4.78) [median (IQR) = 5 (3, 7)] (*p* = 0.042). Also, the mean (SD) HSI score also significantly decreased from 1.62 (1.56) [median (IQR) = 2 (0, 3)] to 0.62 (0.77) [median (IQR) = 0 (0, 1)] (*p* = 0.016) (Table 2).

#### 3.2.3. Knowledge and Attitudes toward Smoking and Smoking Cessation

The results revealed that the mean (SD) score on knowledge of smoking and smoking cessation significantly increased from 12.53 (1.84) [median (IQR) = 13 (12, 14)] at baseline to 13.53 (1.64) [median (IQR) = 14 (13, 15)] in the fourth week (*p* = 0.039). A similar trend was also observed in evaluating attitudes toward smoking and smoking cessation, with a significant increase in mean (SD) score from 39.16 (3.53) [median (IQR) = 40 (36, 42)] to 40.79 (3.05) [median (IQR) = 40 (39, 43)] (*p* = 0.032) (Table 2, Tables S2 and S3).

#### 3.2.4. Satisfaction and Confidence in Quit with US

After 4 weeks of Quit with US use, the results showed that all 19 participants were satisfied with the overall design with a mean (SD) score of 3.95 (0.84). They also expressed satisfaction toward the overall content with a mean (SD) score of 4.17 (0.81). Furthermore, they expressed confidence in using the smartphone app with a mean (SD) score of 4.28 (0.76) (Table 2, Tables S4 and S5).

## 4. Discussion

### 4.1. Principal Findings

This study demonstrated the use outcomes of a novel smartphone app for smoking cessation specifically developed for young adult smokers. After 4 weeks of study, almost one-third (31.6%) of participants succeeded in quitting smoking measured by the self-reported 7-day point prevalence abstinence and subsequently verified by the level of exhaled CO concentration. The overall exhaled CO concentration level significantly decreased from baseline.

In addition, the participants improved their smoking behaviors as compared with before using Quit with US. The daily cigarette consumption and level of nicotine dependence also significantly decreased. The decline in daily cigarette consumption was consistent with several related studies exploring the use of a smartphone app ranging in periods from 1 to 6 months [22,23,25,27].

Compared with related studies, the abstinence rate at 4 weeks observed in this study was higher than those of studies in the US (2.6%) [25], in Australia (4.5% and 18.9%) [28,44], and in the UK (26.0%) [29] but was lower than that of study in Japan (71.6%) [26] in which the abstinence rate was measured by self-report regardless of the further measurement of exhaled CO concentration level. The variations in abstinence rates might have been due to the differences in terms of inclusion criteria and smoking cessation treatments that participants received when participating in the studies. Considering the inclusion criteria, while this study included participants, who smoked at least one cigarette in the last 30 days, the study in Australia [28,44] included current smokers without any defined frequency of smoking, and other related studies including participants restrictively to those who were daily smokers [25,26]. In addition, this study did not exclude participants who used other tobacco products, while a related study excluded participants who used e-cigarettes [25]. However, our findings indicated that the abstainer and the nonabstainer groups were balanced in the type of tobacco products used at baseline (*p* > 0.05) (Table S6). Also, the abstinence rates could be affected by smoking cessation treatments that participants received other than smartphone apps. While this study and the study in the US [25] provided brief counseling on smoking cessation, the study in Japan [26] provided pharmacotherapy, and other related studies [28,29,44] gave participants the freedom to use other smoking cessation supports.

Three possible reasons could explain the positive outcomes of Quit with US use. Firstly, and most importantly, Quit with US was developed based on the 5 A’s model. The 5 A’s model, comprising five major steps; ask, advise, assess, assist, and arrange, has been widely applied as a recommended method in guidelines for smoking cessation [12,13]. With these five major steps, smokers would be more likely to achieve their goals in quitting smoking compared with those who not receiving the 5 A’s model-oriented support [45]. Quit with US, containing contents applied from the 5 A’s model, passed the evaluation of content analysis assessed by pharmacists specialized in smoking cessation. The smartphone app had also undergone an evaluation of opinions from young adult smokers before further modifying and developing.

Secondly, Quit with US was also developed based on the self-efficacy theory, an effective and well-documented supporting strategy. Self-efficacy is expressed as individuals’ self-confidence in the capabilities to quit smoking and to manage the temptation to resume smoking [33].

Lastly, the smartphone app was designed to be appropriate for young adult smokers as the target users. The finding showed that the participants were satisfied with its overall design and content. The three most rated responses regarding design from participants were, “Illustrations and graphics were attractive, interesting, and luminous,” “Color components on the screen were appropriate and attractive,” and “Menu icons could convey a clear understanding of their functions” (Table S4). Moreover, the participants expressed confidence in using the smartphone app. The three most rated responses regarding individuals’ confidence were, “This smartphone app provided accurate information about smoking cessation,” “This smartphone app was considered a safe smoking cessation method,” and “Using this smartphone app as a supplement to other smoking cessation methods could promote successful smoking cessation” (Table S5).

### 4.2. Limitations

This study encountered six limitations. Firstly, the primary outcome of a 7-day point prevalence abstinence was verified by level of exhaled CO concentration. Even though the measurement of exhaled CO concentration was an immediate, noninvasive, well-established method [12,23,25,26,28], and provided convenience to the research team, it was noticed that the results were less accurate than the analysis of cotinine concentration (plasma, saliva, or urine) as CO breath half-life is approximately 5 to 6 h [46]. However, this study employed a 7-day point prevalence abstinence initially by collecting data from self-reports. Participants’ information on quitting smoking during the previous 7 consecutive days was then verified by the level of exhaled CO concentration for smoking abstainers. Secondly, according to the CO breath half-life, participants who smoked at least one cigarette in the last 30 days were included in this study regardless of exhaled CO concentration level. All participants in this study received brief counseling on smoking cessation. Without the smoking cessation consultation, the abstinence rate may have been lower. Further, this study included 19 participants, including both daily and nondaily smokers. Therefore, generalizability to the daily smoker group may be limited. Fifthly, the study period of 4 weeks might not have been sufficient enough to precisely conclude that participants could successfully quit smoking. Lastly, the developed smartphone app was not designed to keep records on users’ interested features, use frequency, or time spent per access. The lack of information was another complication to identify which features in the smartphone app actually contributed to users’ success with smoking cessation.

### 4.3. Future Direction

We plan to continue improving the smartphone app by applying the positive results from the satisfaction evaluation of the use (Tables S4 and S5) to provide the potential users with a more user-friendly version of the smartphone app. After the revamped version of the smartphone app is developed, its outcomes will be assessed in a longer study period and compared with those of the control group.

## 5. Conclusions

This constituted the first study to assess the use of Quit with US, a smartphone app for young adult smokers to achieve smoking cessation. This novel smartphone app was developed based on the 5 A’s model along with the self-efficacy theory. Quit with US was found to be a potentially effective smartphone app providing smoking cessation assistance for young adult smokers. In addition, it could improve smoking behaviors as well as knowledge and attitudes toward smoking and smoking cessation. Participants also expressed satisfaction and confidence in the smartphone app. A future, definitive, randomized control trial investigating its efficacy is warranted.

## Figures and Tables

**Table 1:** Characteristics of the 19 participants at the time of enrollment.

Characteristics	*n* (%)
**Assigned Sex at Birth**	
Male	13 (68.4)
Female	6 (31.6)
**Age (Years), Mean (SD)**	20.42 (1.46)
**Age Started Smoking (Years), Mean (SD)**	17.00 (2.28)
**Smoking Period (Years), Mean (SD)**	3.42 (1.74)
**Tobacco Products Used during the Past Year ^1^**	
Locally made cigarettes	16 (84.2)
E-cigarettes	14 (73.7)
Imported cigarettes	13 (68.4)
Hand-rolled cigarettes	5 (26.3)
Cigars	2 (10.5)
Water pipe/Baraku	1 (5.3)
Pipes	1 (5.3)
**Frequency of Cigarette Smoking per Week**	
1–2 times	3 (15.8)
3–4 times	1 (5.3)
5–6 times	2 (10.5)
7 times	12 (63.2)
>7 times	1 (5.3)
**Daily Smokers**	
Yes	13 (68.4)
No	6 (31.6)
**Daily Cigarette Consumption**	
≤5	10 (52.6)
6–10	6 (31.6)
≥11	3 (15.8)
median (IQR)	5 (3, 10)
**Nicotine Dependence Level (Heaviness of Smoking Index Score) ^2^**	
Low nicotine dependence (0–2 scores)	15 (79.0)
Moderate nicotine dependence (3–4 scores)	4 (21.0)
median (IQR)	0 (0, 2)
**Readiness to Quit Smoking**	
Within the next 30 days	13 (68.4)
Up to the next 6 months	6 (31.6)
**Past Year Quit Attempt**	
Yes	15 (79.0)
No	4 (21.0)
**Previous Use of Smartphone Application for Smoking Cessation**	
Yes	0 (0)
No	19 (100.0)

SD: standard deviation; IQR: quartile range; ^1^ Participants were allowed to select multiple answers from the list.; ^2^ The scores ranged between 0 and 6: 0–2 (low nicotine dependence), 3–4 (moderate nicotine dependence) and 5–6 (high nicotine dependence).

**Table 2:** Comparison outcomes before and after using Quit with US for 4 weeks of 19 participants.

Outcomes	Use of Quit with US				*p*-Value ^1^
	Before		After		
	Mean (SD)	Median (IQR)	Mean (SD)	Median (IQR)	
**Primary Outcome**					
**Smoking Cessation**					
7-day point prevalence abstinence ^2^, *n* (%)			6 (31.6)		
Exhaled CO concentration level (ppm)	9.47 (4.97)	8 (6, 12)	7.84 (3.53)	8 (4, 11)	0.014
**Secondary Outcomes**					
**Smoking Behavior**					
Daily cigarette consumption	7.05 (5.66)	5 (3, 10)	4.00 (4.80)	3 (0, 5)	0.008
Heaviness of Smoking Index score ^3^	1.16 (1.46)	0 (0, 2)	0.42 (0.69)	0 (0, 1)	0.010
**Smoking Behavior of Nonabstainers (*n* = 13)**					
Daily cigarette consumption	9.31 (5.48)	7 (5, 10)	5.85 (4.78)	5 (3, 7)	0.042
Heaviness of Smoking Index score ^3^	1.62 (1.56)	2 (0, 3)	0.62 (0.77)	0 (0, 1)	0.016
**Knowledge and Attitudes**					
Scores on knowledge of smoking and smoking cessation ^4^	12.53 (1.84)	13 (12, 14)	13.53 (1.64)	14 (13, 15)	0.039
Scores on attitudes toward smoking and smoking cessation ^5^	39.16 (3.53)	40 (36, 42)	40.79 (3.05)	40 (39, 43)	0.032
**Satisfaction and Confidence ^6^**					
Satisfaction with the overall design			3.95 (0.84)		
Satisfaction with the overall content			4.17 (0.81)		
Confidence in the overall use			4.28 (0.76)		

CO: carbon monoxide; ppm: parts per million; IQR: interquartile range; ^1^ The Wilcoxon signed-rank tests were used to compare differences between 2-related groups.; ^2^ Measured by answering the question “Did you smoke any cigarette in the past 7 days?” and verified with an exhaled CO concentration level (a cut-off level of ≤6 ppm).; ^3^ The scores ranged between 0 and 6, with 6 indicating the highest nicotine dependence level.; ^4^ The scores ranged between 0 and 15, with 15 indicating the highest score on knowledge of smoking and smoking cessation.; ^5^ The scores ranged between 15 and 45, with 45 indicating the highest score on attitudes toward smoking and smoking cessation.; ^6^ The mean scores ranged between 1 and 5, with 5 indicating the highest satisfaction and confidence.

## Data Availability

The data presented in this study are available from the corresponding author on reasonable request.

## Supplementary Materials

The following are available online at https://www.mdpi.com/article/10.3390/ijerph18179376/s1, Table S1: A brief counseling manual for smoking cessation consultation, Table S2: Knowledge of smoking and smoking cessation of 19 participants, Table S3: Attitudes toward smoking and smoking cessation of 19 participants, Table S4: Satisfaction evaluation after using Quit with US of 19 participants, Table S5: Confidence evaluation after using Quit with US of 19 participants, Table S6: Difference in biochemically verified 7-day point prevalence abstinence by type of tobacco products used of 19 participants at baseline.
