# Supplementary material for: Feasibility of a Smoking Cessation Smartphone App (Quit with US) for Young Adult Smokers: A Single Arm, Pre-Post Study
Source: Int J Environ Res Public Health. 2021 Sep 5;18(17):9376. doi: 10.3390/ijerph18179376 (PMC8430656; doi:10.3390/ijerph18179376)
Supplement: Supplementary file 1 [file ijerph-18-09376-s001.zip › ijerph-1267321 - Table S1 - Revised Manuscript (R2).pdf]

Table S1. A brief counseling manual for smoking cessation consultation.

5 A's	Action
**Ask participants about cigarette and tobacco products use.**	Ask participants about cigarette and tobacco products use, for instance, the following.<br>• "Do you smoke cigarettes?"<br>• "What kind of tobacco products do you use?"
**Advise participants to quit smoking.**	Recommend participants to quit in a clear, strong, and personalized manner, for instance, the following.<br>• "It is important that you quit smoking now, and I can help you."<br>• "Quitting smoking is the most important thing you can do to protect your health."<br>• "You can save money by quitting smoking."
**Assess readiness to make a quit attempt and nicotine dependence level.**	Assess readiness to make a quit attempt, for instance, the following.<br>• "Would you like to be a nonsmoker?"<br>• "Do you think you have a chance of quitting successfully?"<br>• "Are you ready to quit smoking now?"
	Assess nicotine dependence level using the Heaviness of Smoking Index (HSI) by asking these two questions.<br>• "How many cigarettes do you smoke daily?"<br>- 1-10 cigarettes (0 score)<br>- 11-20 cigarettes (1 score)<br>- 21-30 cigarettes (2 scores)<br>- ≥31 cigarettes (3 scores)<br>• "How soon after waking up do you smoke your first cigarette?"

	- Within 5 minutes (3 scores)<br>- 6-30 minutes (2 scores)<br>- 31-60 minutes (1 scores)<br>- ≥61 minutes (0 scores)<br>The HSI summed scores are separated in 3 levels of nicotine dependence.<br>• Low nicotine dependence (0-2 scores)<br>• Moderate nicotine dependence (3-4 scores)<br>• High nicotine dependence (5-6 scores)
	Assess causes of cigarette dependence, for instance, the following.<br>• "When do you usually smoke a cigarette?"<br>• "What do you usually do during cigarette smoking?"<br>• "What do you feel when you smoke a cigarette?"
**Assist participants with a quit plan.**	Participants who are ready to quit smoking are assisted in smoking cessation using the STAR technique, for instance, the following.<br>• "Set a target quit date." "The target quit date should ideally be within 2 weeks."<br>• "Tell your families, friends, and coworkers about your decision to quit, and ask for their understanding and support."<br>• "Anticipate challenges to your upcoming quit attempt, especially during the critical first few weeks, including nicotine withdrawal symptoms, using the 5D's."<br>- Delay: "Delay until the craving to smoke has passed."<br>- Deep breath: "When you feel the urge to smoke, try breathe deeply."

	- Drink water: "Drinking plenty of water." "Sipping water when urged to smoke a cigarette."<br>- Do something else: "Distract yourself from thoughts of smoking by doing something else."<br>- Destination: "Keep reminding yourself that you would like to quit successfully."<br>• "Remove all tobacco-related products from your home and make it smoke free." "Stay away from places where you use to smoke."
	Participants who were not ready to quit smoking were assisted in smoking cessation using the 5R's.<br>• Relevance: Indicate how smoking is relevant to personal health problems.<br>• Risks: Identify potential negative consequences of smoking that are relevant to personal health problems.<br>• Rewards: Emphasize personal benefits of quitting smoking.<br>• Roadblocks: Determine barriers to quitting and devise a solution.<br>• Repetition: Repeat the motivation to quit at every visit.
**Arrange for follow-up**	Arrange for follow-up at 1, 2, and 4 weeks.<br>• Identify encountered problems.<br>• Anticipate challenges in the immediate future.<br>For participants who can quit smoking<br>• Congratulate and affirm decision to quit.<br>• Offer ongoing encouragement after quitting.<br>For participants who cannot quit smoking or relapse.<br>• Enhance motivation to quit using the 5R's.

	• Explore reasons for relapse, resolve and prevent for future lessons.<br>• Set a new target quit date.
