# Supplementary material for: Feasibility of a Smoking Cessation Smartphone App (Quit with US) for Young Adult Smokers: A Single Arm, Pre-Post Study
Source: Int J Environ Res Public Health. 2021 Sep 5;18(17):9376. doi: 10.3390/ijerph18179376 (PMC8430656; doi:10.3390/ijerph18179376)
Supplement: Supplementary file 1 [file ijerph-18-09376-s001.zip › ijerph-1267321 - Table S2 - Revised Manuscript (R2).pdf]

Table S2. Knowledge of smoking and smoking cessation of 19 participants.

	**Participants replied correct answers, *n* (%)**		***p*-Value [1]**
	**Before use Quit with US**	**After use Quit with US**	
1. Cigarettes are considered one type of addictive substances.	17 (89.5)	19 (100.0)	0.157
2. Nicotine in cigarettes makes smokers addicted to cigarettes.	19 (100.0)	19 (100.0)	-
3. Tar in cigarette contributes to chronic cough, shortness of breath, and lung cancer among smokers.	19 (100.0)	19 (100.0)	-
4. Toxins in cigarette smoke are harmful to both smokers and nonsmokers.	19 (100.0)	19 (100.0)	-
5. Passive smokers obtain fewer toxins in cigarette than active smokers. [2]	13 (68.4)	17 (89.5)	0.102
6. Smoking causes hair loss, premature greying of hair, and wrinkled faces leading to premature aging.	18 (94.7)	19 (100.0)	0.317
7. Smoking causes gum disease, discolored teeth, and bad breath.	19 (100.0)	19 (100.0)	-
8. Smoking causes sexual dysfunctions.	18 (94.7)	18 (94.7)	-
9. Smoking increases risks of heart attack, acute stroke, and paralysis.	16 (84.2)	17 (89.5)	0.564
10. Smoking cessation counseling by specialists, namely, physicians and pharmacists, together with proper	11 (57.9)	11 (57.9)	1.000

medication is considered the most successful means in quitting smoking.			
11. Smokers attempting to quit smoking may experience irritability, distraction, depression, or constipation.	15 (79.0)	15 (79.0)	1.000
12. Smokers attempting to quit smoking may experience increased appetite and weight gain.	11 (57.9)	13 (68.4)	0.317
13. Possible symptoms after quitting smoking will gradually decrease and finally disappear.	17 (89.5)	19 (100.0)	0.157
14. Cigarettes can be sold or given to those under 20 years of age. [2]	14 (73.7)	19 (100.0)	0.025
15. Universities are smoke-free areas.	12 (63.2)	14 (73.7)	0.414

[1] The McNemar test was used to compare proportions between 2-related groups.

[2] Negative-meaning questions
