# Supplementary material for: Feasibility of a Smoking Cessation Smartphone App (Quit with US) for Young Adult Smokers: A Single Arm, Pre-Post Study
Source: Int J Environ Res Public Health. 2021 Sep 5;18(17):9376. doi: 10.3390/ijerph18179376 (PMC8430656; doi:10.3390/ijerph18179376)
Supplement: Supplementary file 1 [file ijerph-18-09376-s001.zip › ijerph-1267321 - Table S3 - Revised Manuscript (R2).pdf]

Table S3. Attitudes toward smoking and smoking cessation of 19 participants.

	Before use Quit with US				After use Quit with US				*p*-Value [2]
	**Agree, *n* (%) [1]**	**Not certain, *n* (%) [1]**	**Disagree, *n* (%) [1]**	**Mean**	**Agree, *n* (%) [1]**	**Not certain, *n* (%) [1]**	**Disagree, *n* (%) [1]**	**Mean**	
1. Smoking one cigarette a day is not harmful to the body. [3]	0	8 (42.1)	11 (57.9)	2.58	1 (5.3)	7 (36.8)	11 (57.9)	2.53	0.954
2. Cigarette smoking creates advantages rather than disadvantages. [3]	2 (10.5)	2 (10.5)	15 (79.0)	2.68	0	1 (5.3)	18 (94.7)	2.95	0.084
3. Smokers tend to have a shorter lifespan compared with nonsmokers.	14 (73.7)	4 (21.0)	1 (5.3)	2.68	18 (94.7)	0	1 (5.3)	2.89	0.172
4. Cigarette smoking causes smokers to possess a negative personality.	13 (68.4)	5 (26.3)	1 (5.3)	2.63	15 (79.0)	3 (15.8)	1 (5.3)	2.74	0.618

5. If you are in a group of smokers, you also have to smoke. [3]	4 (21.0)	6 (31.6)	9 (47.4)	2.26	1 (5.3)	3 (15.8)	15 (79.0)	2.74	0.005
6. Cigarette smoking contributes to higher self-confidence. [3]	1 (5.3)	5 (26.3)	13 (68.4)	2.63	0	4 (21.0)	15 (79.0)	2.79	0.180
7. Cigarette smoking is common among ordinary people. [3]	7 (36.8)	7 (36.8)	5 (26.3)	1.89	5 (26.3)	6 (31.6)	8 (42.1)	2.16	0.046
8. Cigarette smoking behaviors should not be imitated.	18 (94.7)	1 (5.3)	0	2.95	16 (84.2)	1 (5.3)	2 (10.5)	2.74	0.291
9. Cigarette smoking annoys other people.	16 (84.2)	2 (10.5)	1 (5.3)	2.79	17 (89.5)	0	2 (10.5)	2.79	1.000
10. Cigarette smoking is harmful to people nearby.	18 (94.7)	1 (5.3)	0	2.95	18 (94.7)	0	1 (5.3)	2.89	0.970
11. Cigarette-related expenses should be saved to	18 (94.7)	1 (5.3)	0	2.95	18 (94.7)	1 (5.3)	0	2.95	1.000

be spent on other necessities instead.									
12. You are determined to quit smoking or to persuade your friends who smoke cigarettes to quit smoking.	17 (89.5)	0	2 (10.5)	2.79	17 (89.5)	0	2 (10.5)	2.79	1.000
13. Achievement in quitting smoking is regarded as self-conquest.	17 (89.5)	2 (10.5)	0	2.89	18 (94.7)	1 (5.3)	0	2.95	0.317
14. If smoking cessation is needed, counseling by healthcare professionals is required.	8 (42.1)	8 (42.1)	3 (15.8)	2.26	12 (63.2)	6 (31.6)	1 (5.3)	2.58	0.088
15. All areas in campus should be smoke-free.	9 (47.4)	5 (26.3)	5 (26.3)	2.21	10 (52.6)	5 (26.3)	4 (21.0)	2.32	0.311

[1] Percentages may not total 100 because of rounding.

[2] The Wilcoxon signed-rank tests were used to compare differences between 2-related groups.

[3] Negative-meaning questions.
