# Supplementary material for: Feasibility of a Smoking Cessation Smartphone App (Quit with US) for Young Adult Smokers: A Single Arm, Pre-Post Study
Source: Int J Environ Res Public Health. 2021 Sep 5;18(17):9376. doi: 10.3390/ijerph18179376 (PMC8430656; doi:10.3390/ijerph18179376)
Supplement: Supplementary file 1 [file ijerph-18-09376-s001.zip › ijerph-1267321 - Table S4 - Revised Manuscript (R2).pdf]

Table S4. Satisfaction evaluation after using Quit with US of 19 participants.

	**mean (SD)** [1]
**Satisfaction with the overall design**	**3.95 (0.84)**
1. Illustrations and graphics were attractive, interesting, and luminous.	4.32 (0.88)
2. Component colors on the screen were appropriate and attractive.	4.21 (0.92)
3. Menu icons could convey a clear understanding of their functions.	4.21 (0.85)
4. Font styles and sizes were appropriate and obvious.	4.16 (0.90)
5. Number of menu options were appropriate for use.	4.10 (0.57)
6. Buttons were appropriately arranged and user-friendly.	4.00 (0.67)
7. The position arrangement of the components on the screen was appropriate and attractive.	3.95 (0.62)
8. The smartphone app was user-friendly.	3.58 (0.61)
9. The smartphone app runs smoothly without any technical glitch.	3.05 (0.85)
**Satisfaction with the overall content**	**4.17 (0.81)**
10. Displayed presentation of records on users' progress in smoking cessation.	4.32 (0.82)
11. Displayed encouraging messages about smoking cessation.	4.26 (0.73)
12. Advice on smoking cessation methods.	4.26 (0.73)
13. Suggested coping methods when having symptoms after smoking cessation.	4.21 (0.85)
14. Calculated money saved from smoking cessation.	4.21 (0.79)
15. Self-set goals in quitting smoking.	4.21 (0.79)

16. Information about benefits of smoking cessation to the body.	4.16 (0.96)
17. Information about drawbacks of smoking to the body.	4.16 (0.90)
18. A list of questions and answers provided by pharmacists.	4.10 (0.94)
19. Self-set an initial date for smoking cessation.	4.10 (0.74)
20. Suggested coping methods with a desire to smoke or unintentional smoking.	3.89 (0.81)

[1] Mean score of the satisfaction with smartphone app use ranged between 1 and 5, with 5 indicating the highest satisfaction and confidence.
