# Supplementary material for: Feasibility of a Smoking Cessation Smartphone App (Quit with US) for Young Adult Smokers: A Single Arm, Pre-Post Study
Source: Int J Environ Res Public Health. 2021 Sep 5;18(17):9376. doi: 10.3390/ijerph18179376 (PMC8430656; doi:10.3390/ijerph18179376)
Supplement: Supplementary file 1 [file ijerph-18-09376-s001.zip › ijerph-1267321 - Table S5 - Revised Manuscript (R2).pdf]

Table S5. Confidence evaluation after using Quit with US of 19 participants.

	**mean (SD)** [1]
**Confidence in the overall use**	**4.28 (0.76)**
1. This smartphone app provided accurate information about smoking cessation.	4.68 (0.48)
2. This smartphone app was considered a safe smoking cessation method.	4.53 (0.51)
3. Using this smartphone app as a supplement to other smoking cessation methods could promote successful smoking cessation.	4.47 (0.61)
4. This smartphone app enabled me to receive suggestions and assistance at my convenience.	4.37 (0.60)
5. I would suggest those who desire to quit smoking to use this smartphone app.	4.37 (0.60)
6. This smartphone app was considered a financially favorable method when compared with other smoking cessation methods.	4.16 (0.90)
7. I was confident that this smartphone app would assist me to successfully quit smoking.	3.89 (0.88)
8. This smartphone app made me feel confident of my ability to successfully quit smoking.	3.79 (0.98)

[1] Mean score of the confidence in smartphone app use ranged between 1 and 5, with 5 indicating the highest satisfaction and confidence.
