# Supplementary material for: Feasibility of a Smoking Cessation Smartphone App (Quit with US) for Young Adult Smokers: A Single Arm, Pre-Post Study
Source: Int J Environ Res Public Health. 2021 Sep 5;18(17):9376. doi: 10.3390/ijerph18179376 (PMC8430656; doi:10.3390/ijerph18179376)
Supplement: Supplementary file 1 [file ijerph-18-09376-s001.zip › ijerph-1267321 - Table S6 - Revised Manuscript (R2).pdf]

Table S6. Difference in biochemically verified 7-day point prevalence abstinence by type of tobacco products used of 19 participants at baseline.

Tobacco products used during the past year	Biochemically verified 7-day point prevalence abstinence, *n* (%)		Total (*n* = 19), *n* (%)	*p*-Value [1]
	Abstainer (*n* = 6)	Nonabstainer (*n* = 13)		
**Locally made cigarettes**				
Yes	6 (100)	10 (76.9)	16 (84.2)	0.517
No	0	3 (23.1)	3 (15.8)	
**E-cigarettes**				
Yes	5 (83.3)	9 (69.2)	14 (73.7)	1.000
No	1 (16.7)	4 (30.8)	5 (26.3)	
**Imported cigarettes**				
Yes	3 (50.0)	10 (76.9)	13 (68.4)	0.320
No	3 (50.0)	3 (23.1)	6 (31.6)	
**Hand-rolled cigarettes**				
Yes	0	5 (38.5)	5 (26.3)	0.128
No	6 (100.0)	8 (61.5)	14 (73.7)	
**Cigars**				
Yes	0	2 (15.4)	2 (10.5)	1.000
No	6 (100)	11 (84.6)	17 (89.5)	
**Water pipe/Baraku**				
Yes	1 (16.7)	0	1 (5.3)	0.316
No	5 (83.3)	13 (100.0)	18 (94.7)	
**Pipes**				
Yes	0	1 (7.7)	1 (5.3)	1.000
No	6 (100)	12 (92.3)	18 (94.7)	

[1] The Fisher's exact test was used to compare differences between 2 groups.
